# Effects of Intermixing in Sb_2_Te_3_/Ge_1+*x*_Te Multilayers on the Thermoelectric Power Factor

**DOI:** 10.1021/acsami.3c00869

**Published:** 2023-05-01

**Authors:** Heng Zhang, Majid Ahmadi, Wastu Wisesa Ginanjar, Graeme R. Blake, Bart J. Kooi

**Affiliations:** Zernike Institute for Advanced Materials, University of Groningen, Nijenborgh 4, 9747 AG Groningen, The Netherlands

**Keywords:** pulsed laser deposition, thermoelectric power factor, Sb_2_Te_3_/Ge_1+*x*_Te multilayer, intermixing, EDX mapping

## Abstract

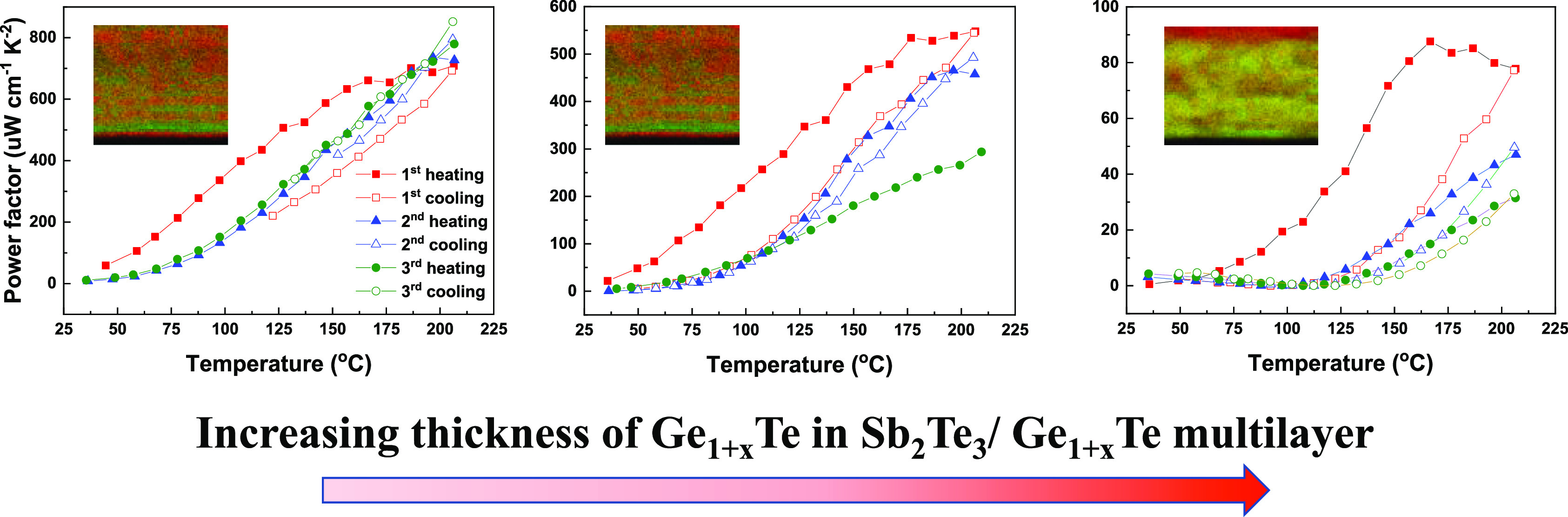

Over the past few decades, telluride-based chalcogenide multilayers, such as PbSeTe/PbTe, Bi_2_Te_3_/Sb_2_Te_3_, and Bi_2_Te_3_/Bi_2_Se_3_, were shown to be promising high-performance thermoelectric films. However, the stability of performance in operating environments, in particular, influenced by intermixing of the sublayers, has been studied rarely. In the present work, the nanostructure, thermal stability, and thermoelectric power factor of Sb_2_Te_3_/Ge_1+*x*_Te multilayers prepared by pulsed laser deposition are investigated by transmission electron microscopy and Seebeck coefficient/electrical conductivity measurements performed during thermal cycling. Highly textured Sb_2_Te_3_ films show p-type semiconducting behavior with superior power factor, while Ge_1+*x*_Te films exhibit n-type semiconducting behavior. The elemental mappings indicate that the as-deposited multilayers have well-defined layered structures. Upon heating to 210 °C, these layer structures are unstable against intermixing of sublayers; nanostructural changes occur on initial heating, even though the highest temperature is close to the deposition temperature. Furthermore, the diffusion is more extensive at domain boundaries leading to locally inclined structures there. The Sb_2_Te_3_ sublayers gradually dissolve into Ge_1+*x*_Te. This dissolution depends markedly on the relative Ge_1+*x*_Te film thickness. Rather, full dissolution occurs rapidly at 210 °C when the Ge_1+*x*_Te sublayer is substantially thicker than that of Sb_2_Te_3_, whereas the dissolution is very limited when the Ge_1+*x*_Te sublayer is substantially thinner. The resulting variations of the nanostructure influence the Seebeck coefficient and electrical conductivity and thus the power factor in a systematic manner. Our results shed light on a previously unreported correlation of the power factor with the nanostructural evolution of unstable telluride multilayers.

## 1 Introduction

Thermoelectric (TE) technology, particularly when exploiting waste heat, has drawn much attention as a potential method to contribute to the global need for energy savings and for power generation.^1^ The efficiency of TE materials is determined by the dimensionless figure of merit (*ZT* = σ*S*^2^*T*/κ), where σ is the electrical conductivity, *S* is the Seebeck coefficient, *T* is the absolute temperature, and κ is the thermal conductivity. A high power factor (PF = σ*S*^2^) implies large voltage and current, and low κ prevents thermal shorting as demanded for achieving high TE performance.^2^ Unfortunately, these parameters σ, *S*, and κ are strongly coupled with each other and with the electronic structure, and in general, improving one leads to the degeneration of the others.^3,4^

In recent decades, significant improvements toward increasing *ZT* have been realized, which to some extent correct for the negative coupling of the underlying parameters. One approach is the nanostructuring of materials, which is applicable to both films and bulk. Hicks and Dresselhaus theorized in the 1990s that multilevel nanostructures with quantum wells should enhance TE performance due to quantum confinement effects by reducing dimensionality.^5,6^ Also, Venkatasubramanian and Harman pointed out that chalcogenide superlattice (SL) films, which consist of two different materials alternatingly stacked on top of each other, exhibited high TE performance mainly because of the reduction in lattice thermal conductivity.^7−9^ These reports pushed toward the investigation of multilayers in applications, such as small-scale coolers and power converters, and soon after, this concept of nanostructuring was utilized in bulk materials giving rise to hierarchical structures.^10^

Multilayer structures have been proven to be beneficial for improvement of the Seebeck coefficient caused by an increase of the density of states (DOS) near the Fermi energy, as well as for reducing the thermal conductivity by the enhancement of phonon confinement and phonon scattering at the interfaces in many systems (but without too much deterioration of the electrical conductivity). For instance, both PbTe/Eu*_x_*Pb_1–*x*_Te multilayers and Si/SiGe superlattices (SLs) show an increased PF inside the quantum wells,^11,12^ and optimized growth direction of GaAs/AlAs SLs predicts superior *ZT* values (∼0.41) compared with the values for bulk GaAs.^13^ Furthermore, SL (multilayer) films of oxides, such as Al_2_O_3_/ZnO, LaNiO_3_/SrTiO_3_ and Ca(OH)_2_/Co_3_O_4_, have shown remarkably high *ZT*,^14−17^ even though the values for the oxides themselves are relatively low. Chalcogenide materials, an extensively studied material class, exhibit favorable properties for high TE performance when structured in multilayer, e.g., Bi_2_Te_3_/Sb_2_Te_3_, PbSeTe/PbTe, Sb_2_Te_3_/MoS_2_, and GeTe/Sb_2_Te.^8,18−20^ Among these, the highest *ZT* value of 2.4 at room temperature was obtained in Bi_2_Te_3_/Sb_2_Te_3_. However, such a high *ZT* value has not been reproduced in similar multilayers up to now. In this case, several issues are ambiguous, including a comprehensive study of the structure and thermal stability, which has been questioned and discussed elsewhere.^10,21−23^ These observations motivate the study of the stability of multilayers and underscore the importance of revealing the structure–property relationship in TE multilayers.

Among the different chalcogenide multilayers, Sb_2_Te_3_/GeTe has hardly been studied in the TE field, although both Sb_2_Te_3_ and GeTe themselves are promising TE materials.^24^ This is probably because Sb_2_Te_3_ and GeTe have a strong tendency to intermix and form GeSbTe compounds. Recently, we used scanning transmission electron microscopy (STEM) to prove that Sb_2_Te_3_/GeTe multilayers deposited at 230 °C consist of alternating Sb_2_Te_3_ and GeSbTe, and after heating at 400 °C, the SL film reconfigures into highly textured bulk GeSbTe.^25^ To study the effect of intermixing on the PF, we have grown (at 210 °C) Sb_2_Te_3_ and Ge_1+*x*_Te films and Sb_2_Te_3_/Ge_1+*x*_Te multilayers with different thicknesses and subjected them to thermal cycling between room temperature and 210 °C. For the Sb_2_Te_3_/Ge_1+*x*_Te multilayers, the in-plane Seebeck coefficient and the corresponding electrical conductivity change substantially after the first heating cycle and then become stable over the next two heating and cooling cycles. It can be deduced that the as-deposited multilayers are not stable and that Sb_2_Te_3_ and Ge_1+*x*_Te intermix (more) during the first heating cycle. In order to study the structure of the actual multilayers, here, we characterize as-deposited and cycled films by atomic resolution and elemental mapping STEM. The images show that, in general, the multilayers consist of Sb_2_Te_3_ and various GeSbTe compounds, but the actual structure not only depends on the thermal history but also on the relative thicknesses of the Sb_2_Te_3_ and Ge_1+*x*_Te sublayers. Our studies unveil the relation between the TE properties and nanostructure in the unstable Sb_2_Te_3_/Ge_1+*x*_Te system.

## 2 Results

### 2.1 Sample Structures

**Figure 1:**
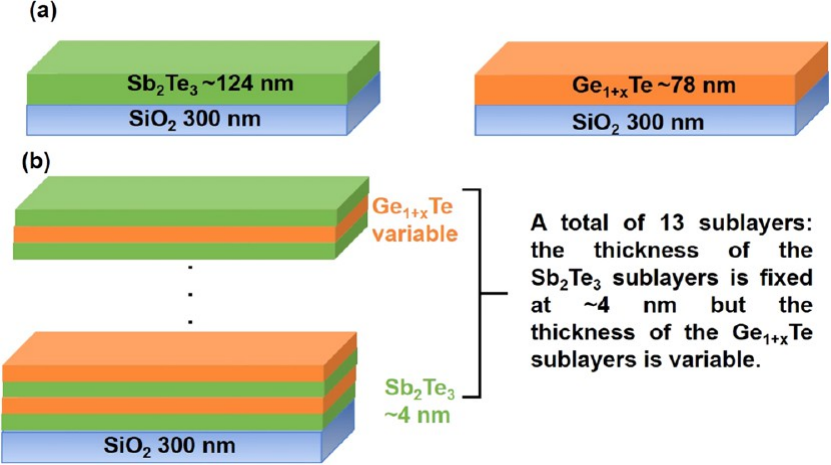
(a) Schematic illustration of the single Sb_2_Te_3_ and Ge_1+*x*_Te films on Si(100) thermal oxide substrates (i.e., Si wafer with a 300 nm amorphous SiO_2_ layer on top). (b) Schematic illustration of the Sb_2_Te_3_/Ge_1+*x*_Te multilayers.

For the single layers of Sb_2_Te_3_ and Ge_1+*x*_Te, films with 10,000 pulses were deposited at 210 °C, yielding thicknesses of ∼124 nm and ∼78 nm, respectively, as measured by atomic force microscopy (AFM) on scratches through the film (see the Supporting Information, Figure S1; the thickness values are averages of three measurements). For the deposition of the multilayers, the first 300 pulses of a Sb_2_Te_3_ film (∼4 nm) were deposited as the first sublayer, and holding the temperature at 210 °C, the following sublayers were deposited in the order Ge_1+*x*_Te and Sb_2_Te_3_ with a repetition of six times. To study the influence of different thickness ratios between sublayers, the Ge_1+*x*_Te sublayers were deposited with either 300, 600, or 1200 pulses, while the Sb_2_Te_3_ sublayers were kept constant (300 pulses), yielding an overall thickness of ∼36, ∼46, and ∼68 nm, respectively, as measured by AFM in the same way as for the monolithic films and also confirmed by STEM images. Then, the thickness of each Ge_1+*x*_Te sublayer in the multilayers is ∼1.3, ∼3, and ∼6.7 nm by calculation. All substrates are oxidized silicon with 300 nm of insulating SiO_2_ on top to avoid the influence of Si. The sample structures are presented schematically in Figure 1. The growth details can be found in the Experimental Section. For simplicity, we refer to the three types of multilayer samples we produced as the “thin,” “medium,” and “thick” samples since the only distinguishing factor is the relative thickness of the Ge_1+*x*_Te sublayers.

### 2.2 Structure and Thermoelectric Properties of Sb_2_Te_3_ and Ge_1+*x*_Te Films

**Figure 2:**
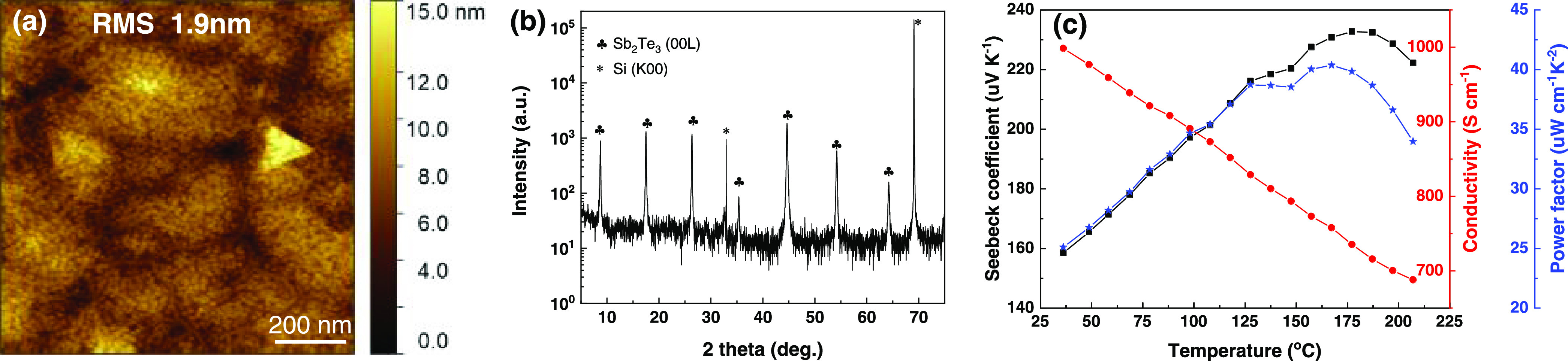
Results for a 124 nm thick Sb_2_Te_3_ film grown using PLD: (a) AFM topography image; (b) θ–2θ XRD scan; and (c) Seebeck coefficient, electrical conductivity, and corresponding power factor as a function of temperature.

Highly textured chalcogenide films (having domains with exclusive (00*l*) out-of-plane orientation and random in-plane orientation) were recently proven to have superior thermoelectric performance.^26,27^ Therefore, we concentrate here on Sb_2_Te_3_ and Ge_1+*x*_Te films and multilayers with (00*l*) out-of-plane orientation to achieve high power factors. Figure 2a shows the surface morphology of a Sb_2_Te_3_ film grown on a thermal silicon oxide substrate using 10,000 PLD pulses. Even though the domains have random in-plane orientation, we still observe triangular facets that originate from domains of rhombohedral Sb_2_Te_3_ having their c-axis out-of-plane.^28,29^ The root-mean-square (RMS) roughness of the ∼124 nm thick Sb_2_Te_3_ film was determined to be ∼1.9 nm, slightly higher than ∼1.2 nm measured for a ∼32 nm thick Bi_2_Te_3_ film on the same substrate.^27^ The out-of-plane texture of the as-deposited Sb_2_Te_3_ film was investigated by performing a θ–2θ X-ray diffraction (XRD) scan, where only (00*l*) Sb_2_Te_3_ peaks are present apart from Si substrate peaks, as shown in Figure 2b. The reflective high-energy electron diffraction (RHEED) pattern (Figure S2b) shows typical streaks, which confirm the relatively smooth surface and domains with random in-plane orientation, similar to the Bi_2_Te_3_ film deposited by the same method.^27^ The temperature-dependent Seebeck coefficient, electrical conductivity, and power factor are presented in Figure 2c. The positive values of the Seebeck coefficient confirm the p-type conductivity of the film. With increasing temperature, the electrical conductivity of the film decreases, indicating a typical metallic-like behavior. However, the Seebeck coefficient increases to ∼233 μV K^–1^ with increasing temperature up to ∼180 °C and then decreases for higher temperatures. The calculated power factor follows the same trend, reaching a maximum of ∼40 μW cm^–1^ K^–2^ at a temperature of ∼170 °C. Both values of the Seebeck coefficient and power factor are close to those measured for a film with the same structure as grown by sputtering and are much higher than the values reported for ordinary (nontextured) Sb_2_Te_3_ films.^26^

**Figure 3:**
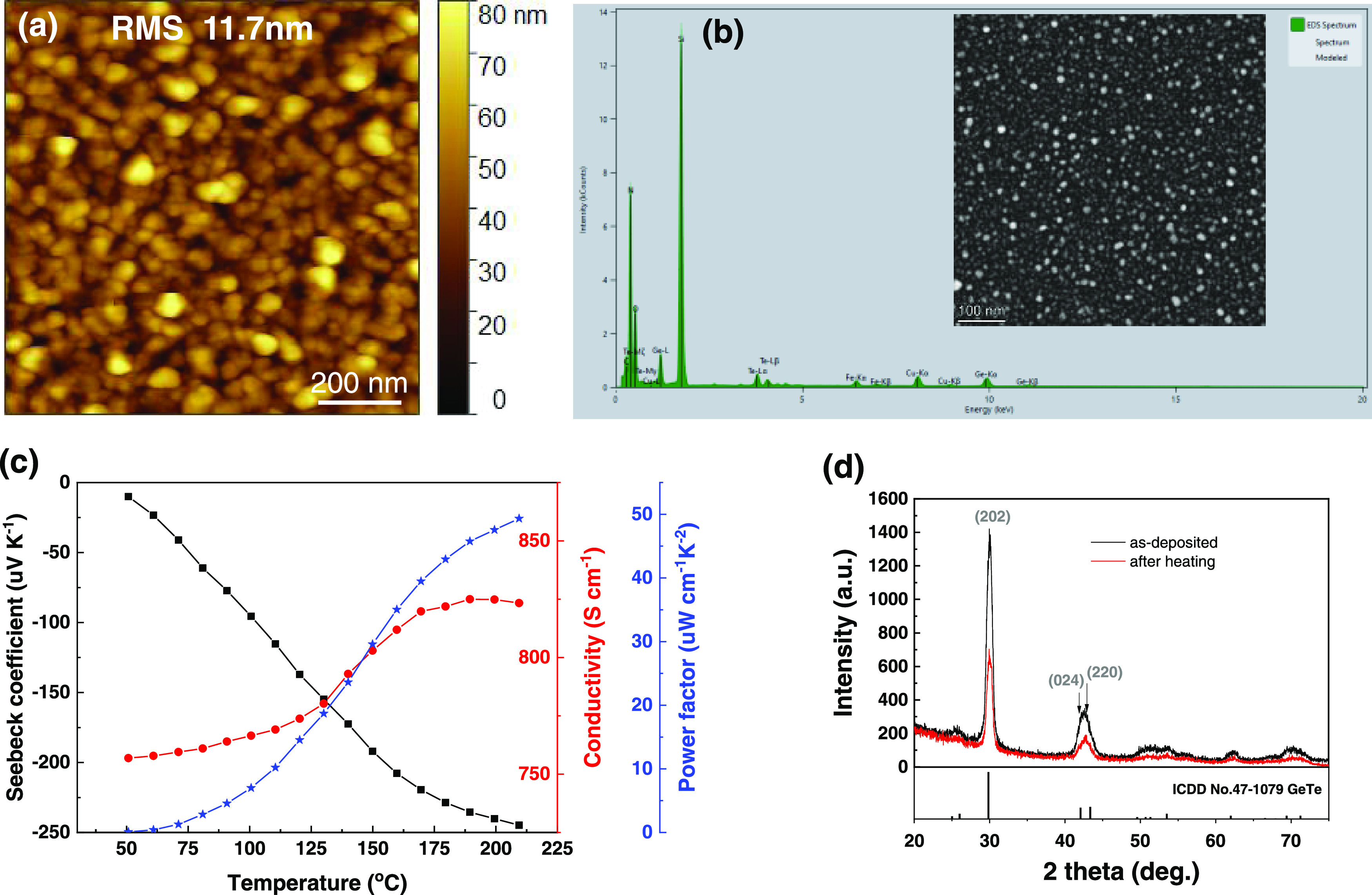
(a) AFM image of the as-deposited Ge_1+*x*_Te film. (b) EDX fitting and HAADF-STEM image of the as-deposited Ge_1+*x*_Te film on silicon nitride grid. Note that, Fe and Cu signals are spurious X-rays from the surrounding and not from the sample itself. (c) Seebeck coefficient, electrical conductivity, and corresponding power factor of the Ge_1+*x*_Te film as a function of temperature. (d) Grazing incidence X-ray diffraction (GIXRD) patterns for the as-deposited Ge_1+*x*_Te film and for the same film after heating. For comparison, the ICDD PDF data for stoichiometric GeTe powder diffraction is shown at the bottom.

As Ge_1+*x*_Te is a three-dimensional (3D)-bonded material, it is not possible to grow a relatively thick film with a dominant c-axis out-of-plane texture on an amorphous substrate. Here, we use a polycrystalline Ge_1+*x*_Te film with the same deposition method to assess the properties of such a film (which is not fully representative of the more textured sublayers used in the superlattice-like films below). The morphology and film composition were investigated by AFM and TEM equipped with an energy-dispersive X-ray spectroscopy (EDX) detector. Figure 3a depicts an AFM image of the as-deposited Ge_1+*x*_Te film surface. When the film is polycrystalline (confirmed by rings in the RHEED pattern, see Figure S2c), it was found that even when the film thickness is clearly lower than that of the two-dimensional (2D)-bonded Sb_2_Te_3_ film (∼78 versus ∼124 nm), the roughness is ∼6 times higher. This is because domains with different orientations gather together forming spherical-like clusters. In order to investigate the atomic structure and the chemical composition of the Ge_1+*x*_Te film, high-angle annular dark-field scanning transmission electron microscopy (HAADF-STEM) in combination with energy-dispersive X-ray spectroscopy (EDS) analysis was performed, and some exemplary results are shown in Figure 3b. To accurately measure the Ge content, a flake of GeTe was exfoliated from the target and used as a reference (see Figure S3, where the target composition was found to be Ge_48.2_Te_51.8_). Interestingly, with relatively low fluence (∼0.78 J cm^–2^), the composition of the film was measured to be Ge-rich (Ge_55.5_Te_44.5_). Note that we can produce the expected Te-rich films with a composition close to that of the GeTe target when the window in the PLD system is fully cleaned (see Figure S4, where Ge_47.2_Te_52.8_ was grown on a SiN grid). However, after one week of use when the window becomes less transparent, and thereby, the fluence reduces, the films turn into Ge-rich. In the present work, we consistently used these Ge-rich films and sublayers.

The Seebeck coefficient, electrical conductivity, and power factor of the Ge_1+*x*_Te film are shown in Figure 3c. In general, GeTe is a nonstoichiometric compound, in which typically 2–3% Ge vacancies are present, generating a high concentration of free charge carriers (∼10^20^–10^21^ cm^–3^ holes) and thereby forming a (degenerate) p-type semiconductor.^30−32^ In the case of our Ge_1+*x*_Te film, however, the negative Seebeck coefficient shows that the film is an n-type semiconductor, which probably originates from the excess Ge in the film. This excess Ge in the Ge_1+*x*_Te film annihilates the Ge vacancies and produces free electrons.^33^ Such an excess Ge composition in PLD-grown GeTe films was also reported before.^34^ According to the theoretical calculation, n-type GeTe has better thermoelectric properties than p-type GeTe.^35^ Upon heating, the electrical conductivity and the power factor increase continuously reaching 823 S cm^–1^ and ∼49 μW cm^–1^ K^–2^ at ∼210 °C, respectively. The XRD patterns of the as-deposited and “after heating” Ge_1+*x*_Te films are compared in Figure 3d. The relative intensities of all peaks for each sample are similar to those of the standard rhombohedral GeTe phase. However, their positions are slightly shifted to the right, e.g., 30.0° for the Ge_1+*x*_Te (202) peak compared to 29.85° for the standard GeTe (202) peak. This is consistent with the excess Ge concentration in the Ge_1+*x*_Te films since Ge atoms are smaller than Te atoms. Ge then dissolves in the Ge_1+*x*_Te compound instead of forming a separate Ge phase. From these results, it can be understood that the film is n-type. After heating, the peak intensities of the film drastically decrease without the emergence of any new peak(s) and with no peak shifts. This indicates that the film evaporates during heating. Hence, in the subsequent multilayer depositions, all of the films were capped with ∼4 nm Sb_2_Te_3_ sublayers on top.

### 2.3 Thermoelectric Properties of Sb_2_Te_3_/Ge_1+*x*_Te Multilayers

**Figure 4:**
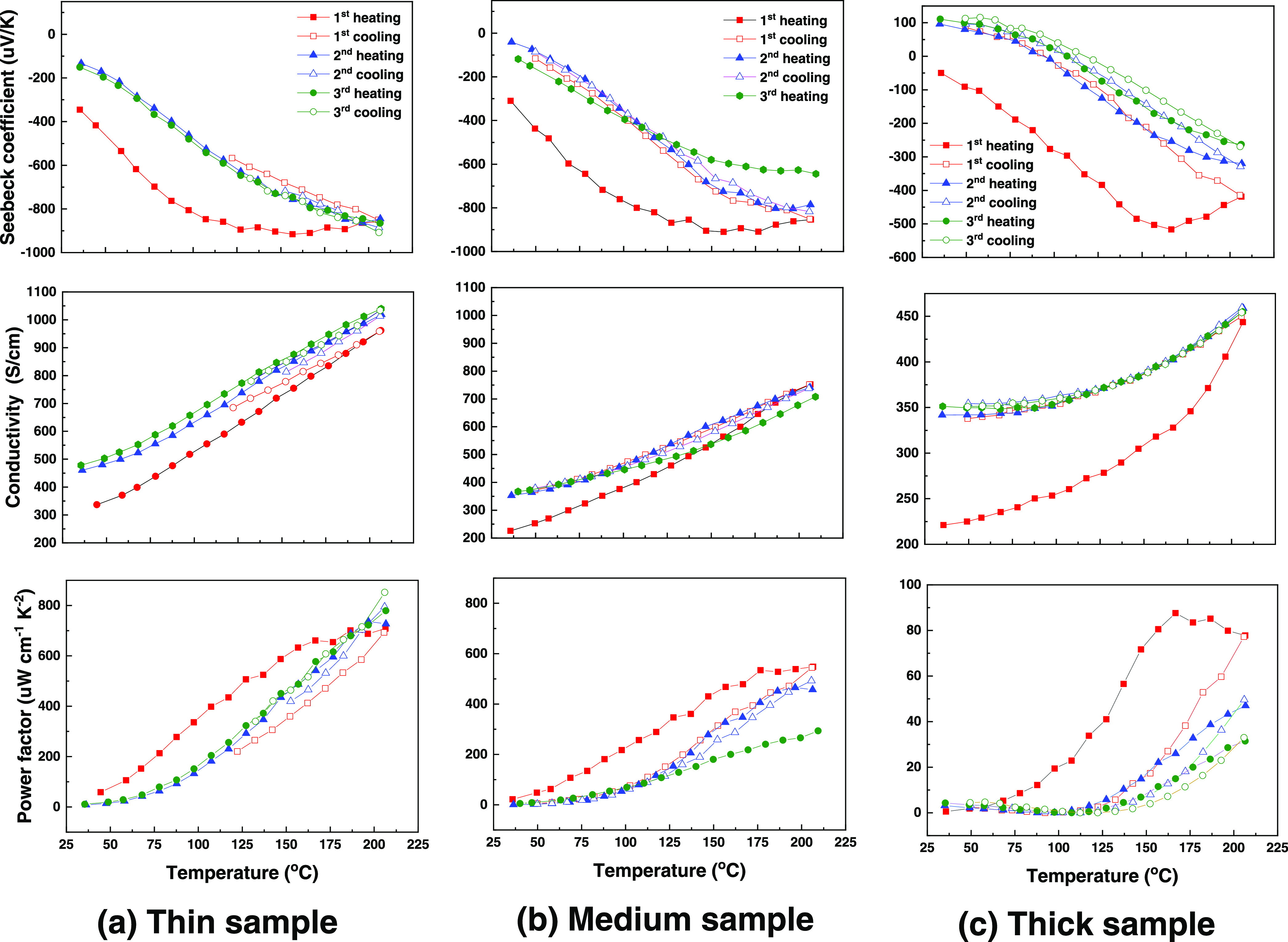
Seebeck coefficient, electrical conductivity, and the resulting power factor of (a) thin sample (300 pulses for the Ge_1+*x*_Te sublayers), (b) medium sample (600 pulses for the Ge_1+*x*_Te sublayers), and (c) thick sample (1200 pulses for the Ge_1+*x*_Te sublayers) over three thermal cycles. Note that the same scales are used for the thin and medium samples, but that we zoom into smaller scales for the thick sample to keep its lower values well visible.

The Seebeck coefficient and electrical conductivity of the as-deposited Sb_2_Te_3_/Ge_1+*x*_Te multilayers measured over three thermal cycles between room temperature and ∼210 °C are displayed in Figure 4. Once deposited, all of the samples are tested in an atmosphere of helium to avoid oxidation effects. Note that the same scales are used for the thin and medium samples, but that we zoom into smaller scales for the thick sample to keep its lower values well visible. During the first heating cycle, the Seebeck coefficients of the thin and the medium samples, i.e., with 300 and 600 pulses Ge_1+*x*_Te sublayers, are similar and increase in magnitude continuously with increasing temperature, reaching 900 μV K^–1^ at ∼155 °C. As the temperature continues to increase, the magnitudes slowly decrease. The thick sample is also an n-type semiconductor, but the absolute Seebeck coefficients are of significantly lower magnitude over the entire temperature range. In addition, the temperature at which the magnitude starts to drop increases to ∼167 °C with a more pronounced downtrend at higher temperatures. The reason for the clearly different Seebeck coefficients is related to the intermixing of Sb_2_Te_3_ and Ge_1+*x*_Te during heating as will be discussed in more detail below in Section 3. For the thin sample, the magnitude of the Seebeck coefficient decreases after initial heating and then remains similar in the subsequent heating and cooling cycles, suggesting that the intermixing between Sb_2_Te_3_ and Ge_1+*x*_Te sublayers is complete and that the structure becomes stable after the first heating cycle. In contrast, the Seebeck coefficient still changes between the second and third cycles for the thick sample. Furthermore, below ∼100 °C, the Seebeck coefficient is positive in the second and third cycles, crossing zero to become negative at higher temperatures.

Comparing the electrical conductivities of these three samples, it is found that with increasing temperature, the conductivity of all samples increases, but that with increasing thickness of the Ge_1+*x*_Te sublayers, the multilayer becomes less conductive. The enhanced conductivity in the first thermal cycle is related to the intermixing of Sb_2_Te_3_ and Ge_1+*x*_Te, leading to the formation of GST compounds, which significantly changes the carrier concentration and/or mobility. Particularly, for the thick sample, this effect is very pronounced during initial heating with an upward swing in electrical conductivity above 167 °C, which presumably corresponds to the temperature interval, where significant intermixing occurs.

The measured power factors are relatively low at room temperature but increase dramatically when the temperature increases to 210 °C. Owing to the significantly improved Seebeck coefficient and the maintained high electrical conductivity, the power factors are substantially enhanced for the multilayer samples in comparison to the single-phase Sb_2_Te_3_ and Ge_1+*x*_Te films. The thin sample, which exhibits the best thermal stability, also shows the highest average power factor of 760 μW cm^–1^ K^–2^ at 210 °C. The highest power factors for the medium and thick samples decay after three thermal cycles from 550 to 290 μW cm^–1^ K^–2^ and 88 to 31 μW cm^–1^ K^–2^, respectively. This result demonstrates the importance of the relative thickness of the Ge_1+*x*_Te sublayer compared to that of Sb_2_Te_3_ for the thermoelectric performance of Sb_2_Te_3_/Ge_1+*x*_Te multilayers.

### 2.4 Nanostructure of Sb_2_Te_3_/Ge_1+*x*_Te Multilayers

In this section, we concentrate on the nanostructure of the films by using HAADF-STEM and elemental mapping based on energy-dispersive X-ray spectrometry (EDX) to investigate the atomic structure, the intermixing between Sb_2_Te_3_ and Ge_1+*x*_Te, and the GST phase formation in the films. It should be noted that all films studied here using STEM had been stored (also due to Covid-19 lockdowns) for one and half years in air. The bright spots in atomic-resolution HAADF-STEM images are roughly proportional to *Z*^2^ (with *Z* being the average atomic number of the column imaged).^36^ Thus, the intensity of the Ge (*Z* = 32) atoms is distinguishable from the Sb (*Z* = 51) and Te (*Z* = 52) atoms. In other words, Ge_1+*x*_Te layers are imaged distinctly from Sb_2_Te_3_ layers. In addition, there is a vdW-like gap between each quintuple Sb_2_Te_3_ block, whereas no gaps exist in the more 3D-bonded Ge_1+*x*_Te layers (although also in GeTe, there is bilayer formation, i.e., Peierls-like distortion, with shorter and longer bonds). Combining the HAADF images with EDX mapping, which shows the spatial distribution of the elements, the intermixing behavior between the layers can be determined unambiguously.

**Figure 5:**
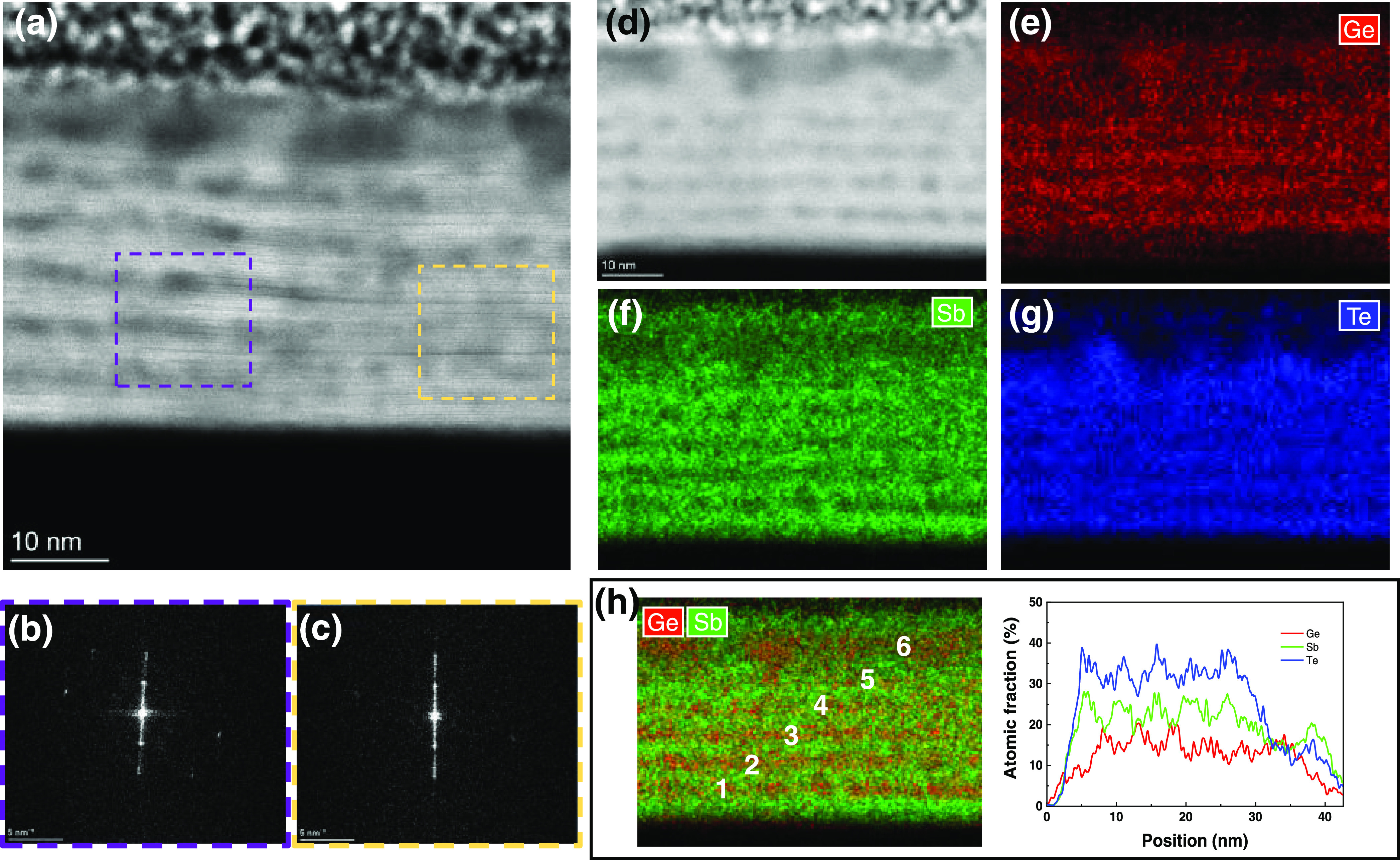
(a) Typical HAADF-STEM image of the as-deposited thin sample. (b,c) FFT patterns of the regions marked by purple and yellow dashed squares in panel (a). (d)–(h) HAADF-STEM image and the corresponding elemental and overlaid EDX mappings of the entire film in the out-of-plane direction. Ge, red; Sb, green; Te, blue. From the relative line profile, the alternating Sb_2_Te_3_ and Ge_1+*x*_Te layers can be discerned, except for the top part (see main text).

Figure 5a shows a typical HAADF-STEM image of the as-deposited thin sample, where six brighter bands corresponding to the Sb_2_Te_3_ sublayers containing thin sharp black lines corresponding to the vdW-like gaps, all more or less parallel to the substrate surface, can be observed. This implies that no severe intermixing occurred between Sb_2_Te_3_ and Ge_1+*x*_Te sublayers during deposition. Furthermore, a slight tilt can be observed between the left and right domains, which is about ∼3° from the fast Fourier transform (FFT) patterns in Figure 5b,c that were obtained from the regions outlined by purple and yellow dashed squares in Figure 5a. In previous reports, a slight tilt in the out-of-plane direction and random in-plane orientation has been proven to substantially improve the power factor.^37^ Furthermore, some “cloudy” dark areas are observed in the film as separated by bright layers. This can be attributed to the Ge_1+*x*_Te sublayers due to the much lower atomic number of Ge atoms. These sublayers do not have a uniform thickness, but Ge_1+*x*_Te shows a clear tendency to form globules. This cloudy characteristic is also visible in Figure 5d, which shows a HAADF image of the region, where the elemental maps depicted in Figure 5e–h are taken. Actually, this characteristic can be found in all of the subsequent STEM results. In the elemental maps and line profile (Figure 5e–h), still rather distinct Ge and Sb layers can be observed although there appears to be some intermixing. Two interesting details can still be observed in these images. First, the Ge-based sublayers are not homogeneous; the positions showing cloudy areas in the HAADF-STEM image (Figure 5d) contain more Ge signal, proving Ge-rich nanoinclusions in the Ge_1+*x*_Te sublayers. Second, the last Ge_1+*x*_Te sublayer (on the top) is thicker than the other Ge_1+*x*_Te sublayers while the penultimate Ge_1+*x*_Te sublayer is only weakly visible. The reason is that, during the 18 months of storage in air, oxygen permeated the Sb_2_Te_3_ capping layer and oxidized the top Ge_1+*x*_Te sublayer, and then drove more Ge atoms from the next Ge_1+*x*_Te sublayer to the top. The oxygen elemental map (see SI Figure S5) is supporting this view, as well as that, Ge_1+*x*_Te is more prone to oxidation than Sb_2_Te_3_. The EDX analysis shows that the overall composition of the film in atomic percentage is Ge/Sb/Te = 20.7:27.2:52.1.

**Figure 6:**
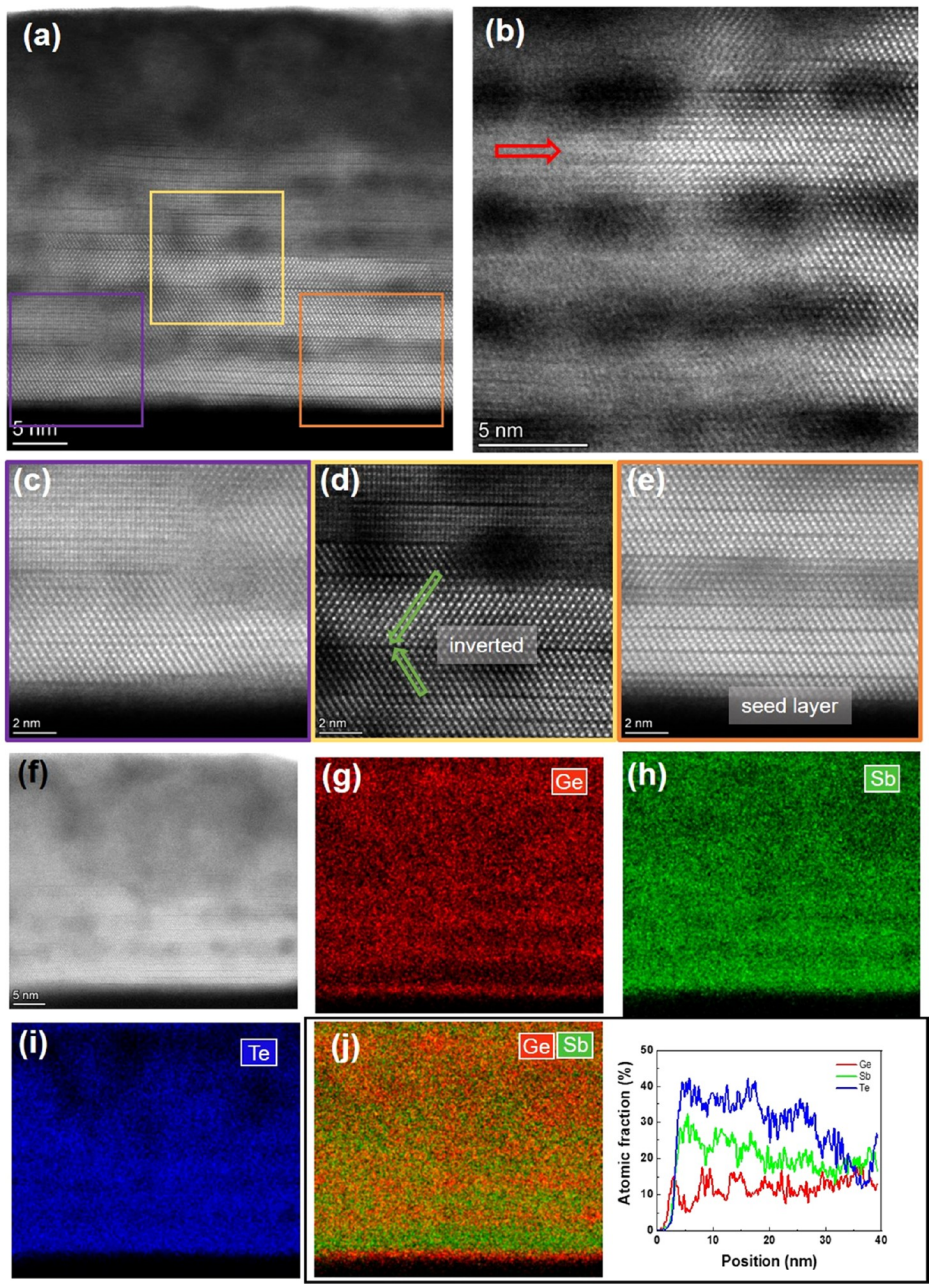
(a) Typical HAADF-STEM image of the thin sample after thermal cycling. (b) HAADF-STEM image from the center of the sample. A remaining single quintuple Sb_2_Te_3_ layer sandwiched between thicker GeSbTe layers is marked by the red arrow, implying that approximately three quintuples have reacted with Ge_1+*x*_Te. (c)–(e) Magnified images of the marked regions in panel (a), where different characteristic features can be observed. GeSbTe blocks with different orientations are shown in panel (c); a 11-layered block in contact with a twinned 17-layered block is shown in panel (d); 4 quintuples of the Sb_2_Te_3_ seed layer can be observed in panel (e). (f)–(j) HAADF-STEM image and the corresponding elemental and overlaid EDX mappings including the entire film in the out-of-plane direction. Ge, red; Sb, green; Te, blue. The relative line profile also suggests the intermixing between Sb_2_Te_3_ and Ge_1+*x*_Te sublayers.

For the thin sample after the three thermal cycles, bright Sb_2_Te_3_ and also GeSbTe containing vdW gaps can be observed in HAADF-STEM images (Figure 6a,b). The red arrow in the center of Figure 6b indicates a single Sb_2_Te_3_ quintuple sandwiched between GeSbTe blocks, illustrating the intermixing of the (about three) outer Sb_2_Te_3_ quintuples with the adjacent Ge_1+*x*_Te sublayers. Enlarged views of the atomic structures for three specific areas indicated in Figure 6a are shown in Figure 6c–e. These results evidence the formation of different GeSbTe structures. Figure 6c shows a region at the bottom of the film, where Sb_2_Te_3_ and Ge_1+*x*_Te sublayers are fully intermixed and GeSbTe blocks are readily observable. From Figure 6d, it is found that due to diffusion, an 11-layered GeSbTe block is in contact with a 17-layered GeSbTe block, and the two adjacent blocks are mutually twinned. This intermixing has also been seen in a previous study on the reconfiguration within GeTe/Sb_2_Te_3_ SLs.^24^ However, in another bottom region, as shown in Figure 6e, four quintuples of the Sb_2_Te_3_ seed layer are still visible. In contrast to the as-deposited sample containing clear layer structures, the EDX mapping and relatively smooth line profile demonstrate the chemical intermixing between the sublayers that occur after thermal cycling, although a weak multilayer structure is still maintained, particularly in the lower part of the film. The top part of the film is affected by oxidation. The elemental ratios (Ge/Sb/Te = 21.0:26.8:52.2) and thickness of this sample are close to those of the as-deposited film, from which we can infer that there is no evaporation during heating. We investigated a “fresh” thin sample after one heating cycle to confirm that the stored samples on which STEM was performed can still represent the structures on which the conductivity and Seebeck coefficient measurements were performed. Indeed, it was found that the atomic structure of a once-heated fresh thin sample exhibited the same structure as the thin sample after the three thermal cycles except for the oxidized top part (see SI Figure S6).

**Figure 7:**
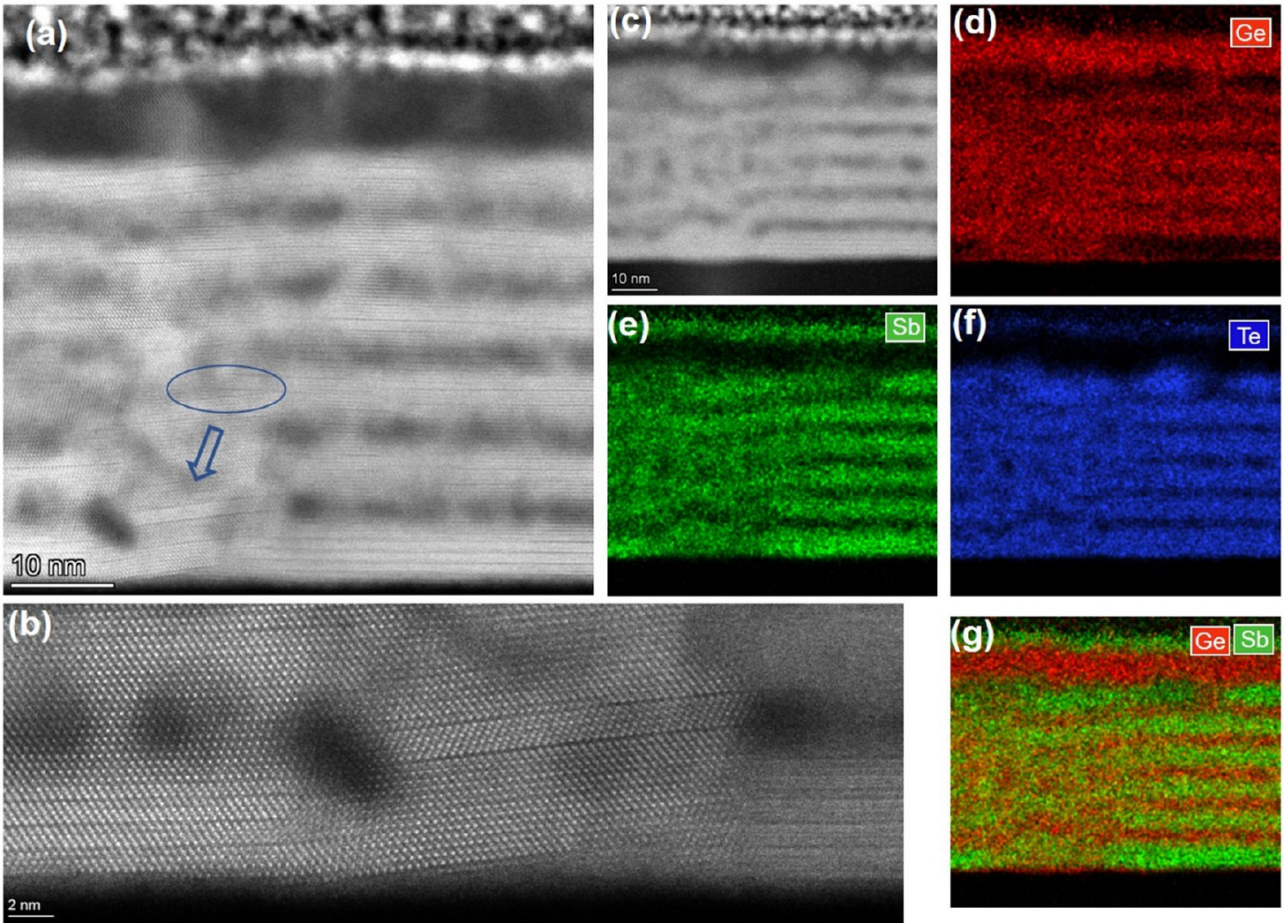
(a) HAADF-STEM image of the medium sample after thermal cycling. A chaotic nanozone arising from intermixing at the boundary between two domains, where GeSbTe blocks are formed with inclined vdW gaps, is indicated by the blue arrow. This inclined region vanishes higher up in the film as indicated by the blue ellipse. (b) Magnified image further showing that this nanozone is completely intermixed and located at a domain boundary. (c)–(g) HAADF-STEM image and the EDX mappings of another region around a domain boundary. Ge, red; Sb, green; Te, blue. It shows that the intermixing behavior can be rather inhomogeneous in different domains.

Next, we use the analogous analysis methods to study the nanostructure of the medium and thick samples after thermal cycling. Figure 7 shows a HAADF-STEM image and the corresponding EDX mappings of the medium sample. Similar features as for the thin sample can be found in this system: The sharp black lines corresponding to the vdW gaps are not continuously parallel to the substrate surface; dark cloudy areas exist in the whole film; and Sb_2_Te_3_ quintuples are still visible (shown in the overview image Figure 7a). However, in this case, we observe more details regarding the intermixing. In a very small local area near the domain boundary, the diffusion of Sb_2_Te_3_ and Ge_1+*x*_Te blocks is more obvious forming a chaotic inclined nanozone, and this tilt ends at an intersection with a horizontal vdW gap, as indicated by the blue arrow and ellipse. In the magnified image, Figure 7b, we can clearly see the Sb_2_Te_3_ blocks at the bottom of each side of the interface. Since the boundary contains more (open space) defects, atoms are able to diffuse more rapidly. More intense diffusion causes a greater degree of atomic rearrangement, including some volume contraction at the boundary, which probably induces the observed tilt. Intriguingly, we also selected this position because it shows that distinctly different intermixing behavior in the sample can be observed across the boundary. The EDX mappings (Figure 7d–g) show rather severe elemental intermixing in the domain on the left side but relatively weak elemental diffusion with a still visible multilayer structure in the domain on the right side.

**Figure 8:**
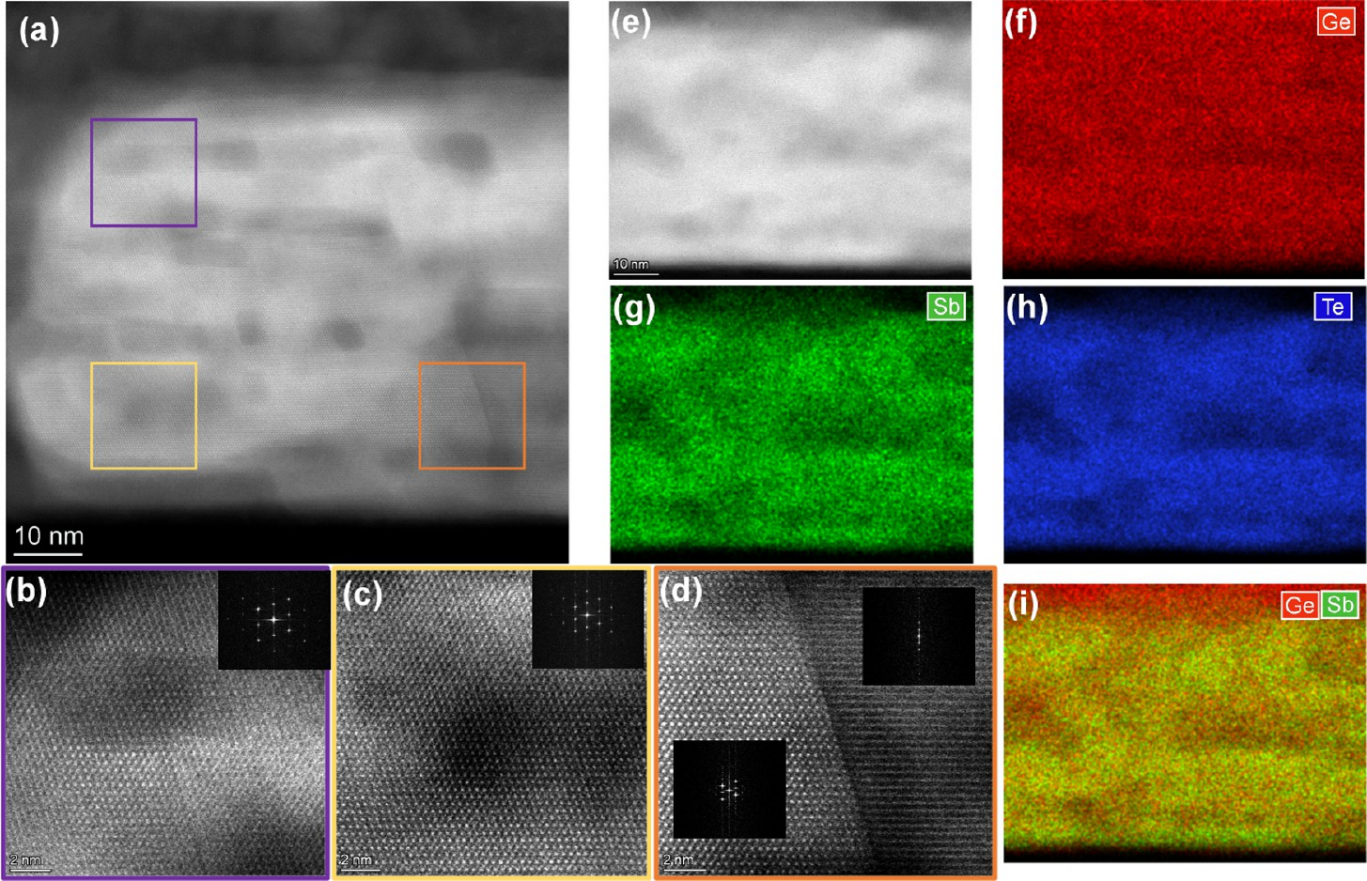
(a) HAADF-STEM image of the thick sample after thermal cycling. (b)–(d) Magnified images of different parts of the film show that intermixing has occurred over the whole film (insets show corresponding FFT patterns). (e)–(i) HAADF-STEM image and the corresponding elemental and overlaid EDX mappings also suggest the full intermixing in the film. Ge, red; Sb, green; Te, blue.

With increasing Ge_1+*x*_Te thickness, the nanostructure of the thick sample after thermal cycling is obviously different. The dark cloudy areas with a higher Ge concentration are still observable in the HAADF-STEM image of the film, as depicted in Figure 8a. Also, domain boundaries are seen in the film, although they are now more sharply connected with crystal facets, where an example is indicated by the orange square. However, no vdW gaps are visible anywhere in the whole film, proving that Sb_2_Te_3_ sublayers have been dissolved within the Ge_1+*x*_Te blocks. Figure 8b–d shows enlarged views of the various colored squares indicated in Figure 8a; the corresponding FFT patterns verify a rhombohedral structure that is very close to cubic, which implies the formation of an overall GeTe-based structure (and not any GST phase-like GST326). Twist domains can be found in Figure 8d. Performing EDX mappings at another position, as shown in Figure 8e–i, suggests that, in agreement with the results of the atomic structure imaging, only Ge-rich nanoinclusions show different contrast, but that overall, the composition is rather homogeneous. Any sign of a multilayer structure has been lost in striking contrast to the thin and medium samples, where after the identical annealing cycles, still the multilayer structure remains visible. Because of drastic solid-state reactions, similar in the annealed Ca(OH)_2_/Co_3_O_4_ multilayers, the volume change induces nanoporosities in this thick sample (not shown here).^17,38^ It is expected that the formation of nanoporosities, similar to Ge-rich nanoinclusions, should reduce the thermal conductivity.

## 3 Discussion

Systematic HAADF-STEM and EDX studies performed in the present work show that the as-deposited Sb_2_Te_3_/Ge_1+*x*_Te multilayer has a clear multilayer structure comprised of Sb_2_Te_3_ quintuples (separated by vdW-like gaps), 3D-bonded Ge_1+*x*_Te layers, and a slight degree of intermixing between the two phases. In contrast, more obvious intermixing of Sb_2_Te_3_ and Ge_1+*x*_Te occurs after thermal cycling even though the maximum temperature reached during cycling was the same as the deposition temperature, i.e., 210 °C. The different arrangements of the layers with, for example, reconfiguration of the vdW gaps, particularly leading initially to GST blocks, are a consequence of the mixing processes that occur during the phase changes associated with the thermal cycling. The formation of the GeSbTe structures is fully consistent with previous reports about intermixing in the GeTe/Sb_2_Te_3_ SL.^24^ However, in the present work, four rather new phenomena are observed.

First, the GeSbTe layers formed during thermal cycling are not always parallel to the substrate surface and the degree of intermixing clearly varies at different locations. Neither effect is observed in the epitaxial SL. This is correlated with the presence of domains with random in-plane orientations and abundant defects within the domains, particularly near domain boundaries. Most likely, Sb and Ge atoms diffuse faster at the boundaries, leading to easier intermixing with a greater degree of atomic rearrangements and involving some volume contraction. These effects give rise to some tilting of the newly formed GeSbTe layers near the domain boundaries. Variability in the detailed structure of the domains, e.g., type and density of defects, will also result in various degrees of intermixing for different domains as we observed experimentally. Analogous behaviors, i.e., faster diffusion next to defects and higher diffusion rate in the in-plane direction than that of cross-plane direction, were also verified in Bi_2_Te_3_/Sb_2_Te_3_ SLs by *in situ* heating experiments.^39^

Second, it is known from previous work that in order to totally intermix GeTe and Sb_2_Te_3_ sublayers into stable GeSbTe (within a reasonable time frame of the order of an hour), an annealing temperature of about 400 °C is required.^24^ However, in the case of relatively thick Ge_1+*x*_Te sublayers, we also find here for the thick sample that nearly total intermixing occurs on a rather short time frame at a temperature as low as 210 °C. Under identical thermal cycling conditions, a much lesser degree of intermixing is observed for the thin and medium samples. If intermixing would be purely determined by kinetics, then it is obvious that thinner sublayers would intermix more easily. However, this is completely contrary to our observations here, which therefore strongly suggest that during intermixing, it is Sb_2_Te_3_ that is dissolving within the Ge_1+*x*_Te. For the thin sample, the amount of Ge_1+*x*_Te is insufficient and the dissolution saturates quickly during the initial heating. This is why the measured thermoelectric properties are stable for the thin sample but more dynamic for the other two samples, in particular for the thick sample where the multilayer structure is completely lost after cycling to 210 °C.

Third, it is shown that cloudy areas, i.e., Ge-rich nanoinclusions, are present in all samples even after thermal cycling, confirming that Sb_2_Te_3_ prefers to interact with GeTe rather than Ge. Also, the top layer of Ge_1+*x*_Te exhibits aggregation of Ge. A drawback of the multilayer films that we analyzed using our advanced STEM studies is that the top part of each film is rather severely oxidized after prolonged storage in air of the films (before they were FIB cut to prepare cross-sectional TEM samples). Nevertheless, sufficiently large regions lower in the films remain unaffected by oxidation to allow proper analyses and conclusions to be drawn. Also, this is proven by a comparison of the nanostructure of a once-heated fresh thin sample with that of the stored thin sample (after three thermal cycles): compare SI Figure S6 with Figure 6 in the main text. The oxidation in the top part of the film shows that the oxygen concentration is strongly correlated with the Ge concentration (and is not correlated with the concentration of Sb, see SI Figure S7). It can be observed that even when the films are capped with Sb_2_Te_3_, severe oxidation and expansion of the GeTe layer below the cap occurs and some Ge is driven to the top and oxidized there. The strong driving force for Ge oxidation and its diffusion to the outer surface was also observed by us for Ge_2_Sb_2_Te_5_ nanoparticles, where after prolonged exposure to air at room temperature, the particles with an initial homogeneous composition develop a clear Ge-oxide outer shell.^40^

The final nanostructure of Sb_2_Te_3_/Ge_1+*x*_Te multilayers not only depends on the thermal treatment but also on the amount of Ge_1+*x*_Te present. This diverse and, in part, complex structure evolution during heating and cooling cycles in return affects the Seebeck coefficient and electrical conductivity. According to the Ge–Sb–Te ternary phase diagram, Sb_2_Te_3_ doped with Ge-rich composition will form n-type GST compounds.^41^ That is why Sb_2_Te_3_/Ge_1+*x*_Te multilayers are always n-type. Note that the structure of the once-heated fresh thin sample is similar to that of the stored thin sample after three thermal cycles (see SI Figure S6). In the case of the thin sample, therefore, we can deduce that the properties changed only during the first heating cycle because (1) the diffusion pathways are rather short such that the intermixing is quickly completed and (2) the dissolution of Sb_2_Te_3_ in GeTe quickly saturates. It has previously been revealed that a high power factor can be achieved in textured dichalcogenide films along the ab-plane, values of which are 3 times higher than in a bulk ingot and 4 times higher than for a single-crystal-like film.^27^ Due to quantum confinement, SL films exhibit even more superior thermoelectric properties.^5,42^ It is clear to see that the thin and medium samples still consist of trigonal GeSbTe and rhombohedral Sb_2_Te_3_ nanostructures with a c-axis texture and they locally maintain their alternating structure after thermal cycling. Therefore, a high power factor is expected. On the contrary, after thermal cycling, the thick sample forms a uniform GeSbTe single layer with a polycrystalline structure close to that of GeTe. Because of more Ge-rich nanoinclusions and drastic solid-state reactions, the carrier scattering is more intense in the thick sample both before and after thermal cycles leading to the sample exhibiting lower electrical conductivity. Xu et al. found the same rhombohedral phase in GeTe-rich GeSbTe compounds, where annealed compounds exhibited excellent performance due to the migration of Ge vacancies to the long-range defects (analogous to vdW gaps).^43^ On the contrary, the thick sample, however, no longer contains vdW gaps and a multilayer structure after the heating cycles, which leads to a 5- to 10-fold decrease of the power factor. In addition to the loss of the quantum confinement effect, also a lowering of the defect concentration combined with a reduced disorder, e.g, due to stacking faults and Ge/Sb intermixing in trigonal GeSbTe,^44,45^ could be reasons for the lower Seebeck coefficient in the thick sample. The much bigger drop in the Seebeck coefficient of the thick sample between initial heating and cooling is a hint that, once GeSbTe is formed at high temperatures, it is close to a cubic GeTe structure. Interestingly, the Seebeck coefficient values below ∼100 °C change from negative to positive with the increase of the temperature after the initial heating, indicating that the predominant charge carriers change from electrons to holes. The Seebeck coefficient becomes negative again but with a smaller magnitude than in the initial heating cycle above ∼100 °C. As a result, the power factor decreases from 78 to 33 μW cm^–1^ K^–2^ at 210 °C. This n-p switching of the GeSbTe film just by changing the temperature is quite unusual and could be used in potential applications in semiconductor switches or sensors.^46^ Research by Rosenthal et al. also shows a tendency toward n-p switching in Sb_2_Te_3_(GeTe)_19_ ingots.^45^ However, the reason for the current switching in the GeSbTe compound is still unclear. Nilges et al. found p-n-p switching in Ag_10_Te_4_Br_3_ and ascribed this reversal to the reorganization of Te_4_ units accompanied by a change of the electronic band structure.^46^ Such behavior was also observed in AgBiSe_2_ during the phase transition, when the Ag/Bi bimetal exchange occurs via Ag vacancies, changing the density of states (DOS) at the Fermi level.^47^ Obviously, this is not the case for our film, as no phase transition occurs around 120 °C for this crystalline GST. The likely reason is that the doping in this complicated film gives rise to Fermi surface reconstruction as inferred from FeSe_2–*x*_ and its doped variants.^48^

## 4 Conclusions

Highly textured telluride films with (00*l*) crystal plane orientation show superior thermoelectric power factors, which not only apply to single layers but also for multilayers. The nanostructure of Sb_2_Te_3_/Ge_1+*x*_Te multilayers can be tuned by varying the relative thickness of the Ge_1+*x*_Te sublayers and thereby the overall composition after thermal cycling. When the Ge_1+*x*_Te sublayers are thin, dissolution of the Sb_2_Te_3_ into the Ge_1+*x*_Te is limited and also the intermixing is limited to the first heating cycle due to short diffusion pathways. This still yields after three thermal cycles relatively ordered 2D layered structures, and therefore, the high Seebeck coefficient and electrical conductivity of the as-deposited structure are largely maintained after annealing. However, in thick Ge_1+*x*_Te sublayers, Sb_2_Te_3_ can dissolve well. Due to the longer diffusion pathways, intermixing is a continuing process during three heating cycles. This finally leads to relatively homogenous 3D GST compounds with a structure close to that of GeTe, which is in agreement with the persistent variation and general deterioration of the thermoelectric properties that we measure during the three thermal cycles. The maximum temperature reached during thermal cycling was only 210 °C, indicating that the intermixing between Sb_2_Te_3_ and Ge_1+*x*_Te occurs at relatively low temperatures. This is also confirmed by the intermixing that is already observed in the as-deposited multilayer, where a deposition temperature of 210 °C was employed.

We therefore show the effect of the gradual intermixing of Sb_2_Te_3_ and Ge_1+*x*_Te sublayers to GeSbTe alloy on the power factor, where the intermixing is retarded when Ge_1+*x*_Te sublayers get thinner compared to the Sb_2_Te_3_ sublayers. This explains the clear difference in (evolution of the) thermoelectric performance under thermal cycling. To maintain the ordered 2D layered structures, relatively thin Ge_1+*x*_Te sublayers compared to Sb_2_Te_3_ must be used. The unexpectedly low temperatures necessary to induce intermixing strongly imply that to evaluate the properties of these unstable thermoelectric multilayer films, measurements must be performed over several thermal cycles until the nanostructures of these systems become long-term stable.

## 5 Experimental Section

Thin films of Sb_2_Te_3_, GeTe, and Sb_2_Te_3_/Ge_1+*x*_Te multilayers were grown on Si(100) covered with thermal oxide (∼300 nm) substrates by pulsed laser deposition (PLD). The growth was monitored using reflective high-energy electron diffraction (RHEED). The corresponding targets were obtained from KTECH with a purity of 99.999%. The substrate cleaning method is described elsewhere.^49^ For the single layers of Sb_2_Te_3_ and GeTe, first, a “seed” layer of 200 pulses (∼3 nm) film was grown at RT, followed by heating to 210 °C with a heating rate of 10 °C min^–1^. Then, 9800 pulses were applied to deposit films at 210 °C. For the deposition of the multilayers, first, a seed layer of 200 pulses Sb_2_Te_3_ was grown at RT and heated to 210 °C with a heating rate of 10 °C min^–1^, followed by the application of 100 pulses to deposit a Sb_2_Te_3_ film to finalize the first sublayer. Holding at 210 °C, the following sublayers were then deposited in the order Ge_1+*x*_Te followed by Sb_2_Te_3_ with a repetition of six times, where the Ge_1+*x*_Te sublayers were deposited with either 300, 600, or 1200 pulses to obtain three different samples, whereas the Sb_2_Te_3_ sublayers were kept constant (300 pulses). During all of the depositions, we applied a pressure of 0.12 mBar Ar gas with 1 sccm flow, a laser fluence of 0.78 J cm^–2^, and a repetition of 1 Hz. The distance between targets and the substrate was kept at 5.2 cm.

θ–2θ X-ray diffraction (XRD) and grazing incidence X-ray diffraction (GIXRD) were performed by using a Panalytical X′pert Pro diffractometer. To characterize surface morphology and acquire film thickness, samples were measured by atomic force microscopy (AFM) using a Bruker MultiMode 8 and analyzed by Gwyddion software. *In situ* RHEED was used to observe the surface structure of the films during the deposition. We used a focused ion beam (FIB, Helios G4 CX DualBeam) to prepare transmission electron microscopy (TEM) cross-sectional specimens with final ion polishing at 1 kV. High-resolution STEM images and EDS maps were obtained using a probe- and image-corrected Thermo Fisher Scientific Themis Z microscope operating at 300 kV. Seebeck coefficients and electrical conductivity were measured simultaneously using a Linseis LSR-3 apparatus.

## Supporting Information Available

AFM images and the average thicknesses of the single Sb_2_Te_3_ film and the single Ge_1+*x*_Te film (Figure S1); RHEED patterns of thermal oxide Si(100) substrate, single Sb_2_Te_3_ film, single Ge_1+*x*_Te film, and different Ge_1+*x*_Te sublayers on the 300 pulses Sb_2_Te_3_ seed layer (Figure S2); HAADF-STEM image, EDX mapping, and EDX spectrum of GeTe target (Figure S3); TEM and EDX spectrum of the GeTe film, which was deposited just after the PLD system had been cleaned (Figure S4); EDX mapping of the element O in the as-deposited thin sample (Figure S5); HAADF-STEM images and strain analysis by geometric phase analysis (GPA) of a fresh thin Sb_2_Te_3_/Ge_1+*x*_Te multilayer (Figure S6); HAADF-STEM image and corresponding elemental line profiles of the “medium sample” after thermal cycling (Figure S7) (PDF)
