# Supplementary material for: Effects of Intermixing in Sb2Te3/Ge1+xTe Multilayers on the Thermoelectric Power Factor
Source: ACS Appl Mater Interfaces. 2023 May 1;15(18):22672–83. doi: 10.1021/acsami.3c00869 (PMC10176324; doi:10.1021/acsami.3c00869)
Supplement: Supplementary file 1 — am3c00869_si_001.pdf [file am3c00869_si_001.pdf]

(Supporting Information)

Effects of Intermixing in $Sb_2Te_3/Ge_{1+x}Te$ Multilayers on the Thermoelectric Power Factor

Heng Zhang*, Majid Ahmadi, Wastu Wisesa Ginanjar, Graeme R. Blake and Bart J. Kooi*

Zernike Institute for Advanced Materials, University of Groningen, Nijenborgh 4, 9747 AG Groningen, The Netherlands

*Corresponding authors: heng.zhang@rug.nl (H. Zhang); b.j.kooi@rug.nl (B. J. Kooi)

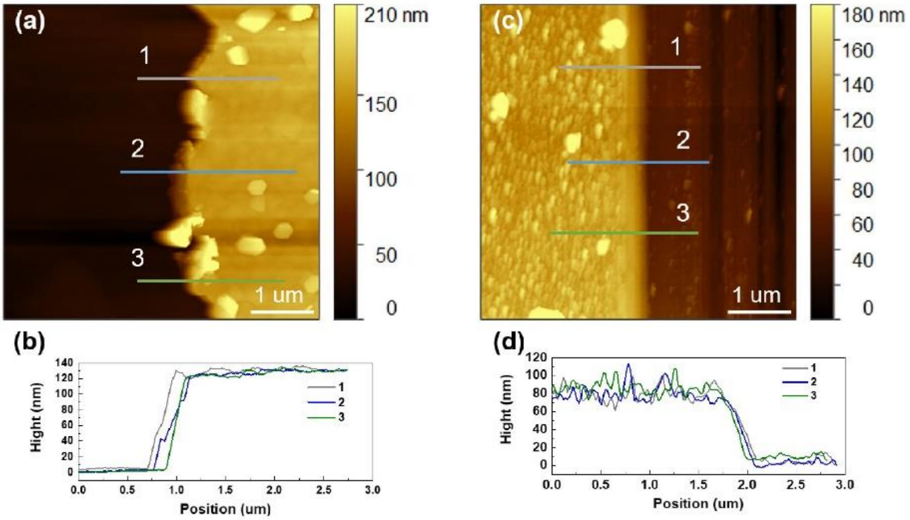

Figure S1: (a) AFM scan showing the edge of a scratch in the Sb_2Te_3 film. The lines show the places where line profiles were taken. The average thickness of the Sb_2Te_3 film determined in this way is ~124 nm as seen in (b). (c) and (d) show the same measurements for the $Ge_{1+x}Te$ film, where the thickness is ~78 nm. It can be observed in the line scans that the surface of the $Ge_{1+x}Te$ film is much rougher than that of the Sb_2Te_3 film.

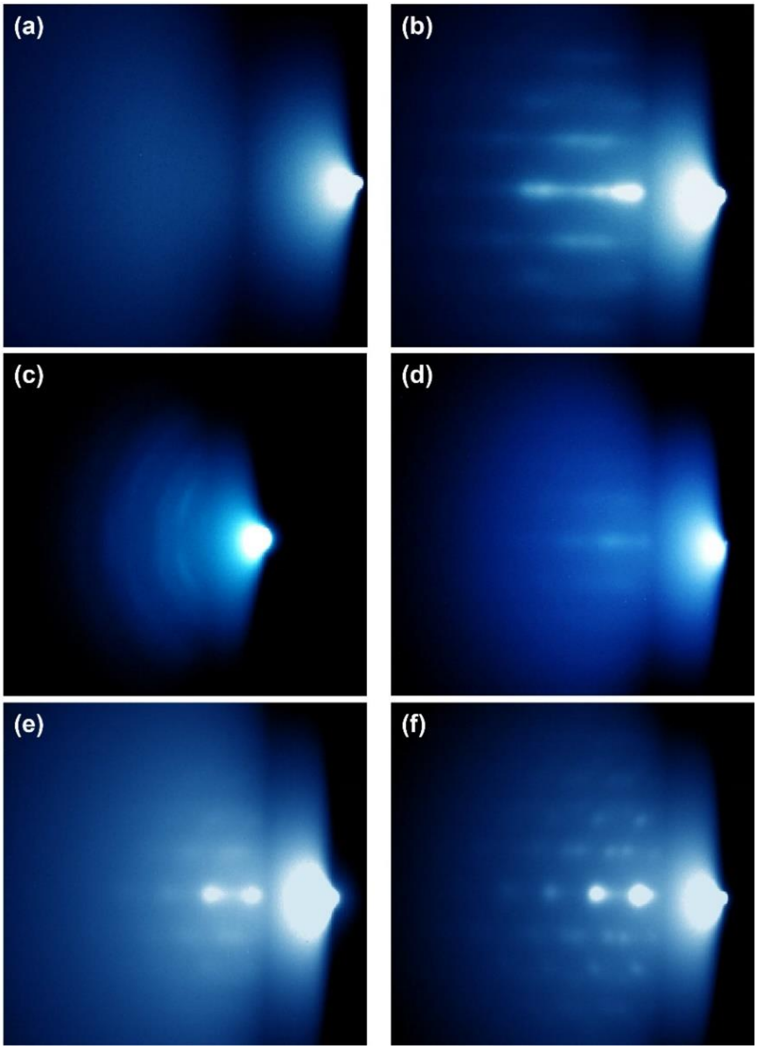

Figure S2: (a) RHEED pattern of bare Si (100) substrate covered with ~300 nm thermal oxide. (b) and (c) RHEED patterns of Sb_2Te_3 film and $Ge_{1+x}Te$ film after 10,000 pulses deposition, respectively. The streaky features show that the as-deposited Sb_2Te_3 film is highly-textured. The ring-shaped patterns indicate that the as-deposited $Ge_{1+x}Te$ film is polycrystalline. (d)-(f) RHEED patterns recorded in-situ after deposition of 300, 600, 1,200 pulses of $Ge_{1+x}Te$ on the 300 pulses Sb_2Te_3 'seed' layer. These films are clearly more textured than the 10,000 pulses film. Only the 300 pulses $Ge_{1+x}Te$ film is still highly textured and smooth. When increasing the film thickness to 600 and 1,200 pulses particularly the surface roughness increases rapidly.

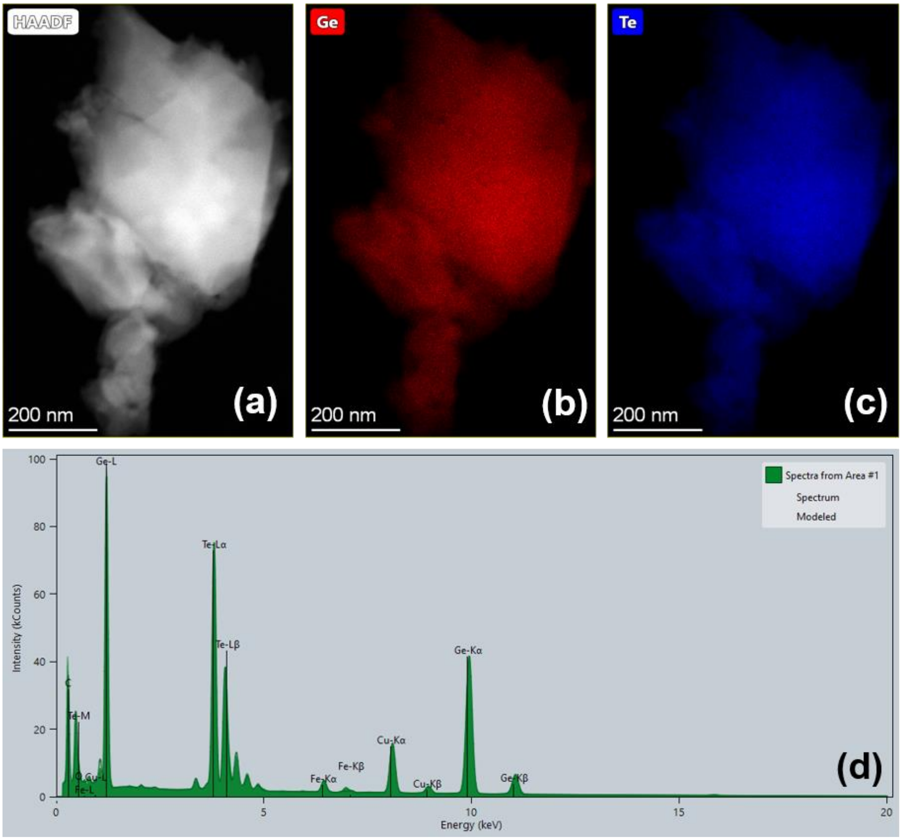

Figure S3: (a) HAADF-STEM image of a GeTe flake exfoliated from the GeTe PLD target. (b) and (c) EDX mapping of the elements Ge and Te, in red and blue respectively. (d) EDX spectrum of the GeTe flake. Quantitative analysis of the spectrum shows that the composition of the target is $Ge_{48.2}Te_{51.8}$. The same quantitative procedure was applied for the analysis of all the (TEM) samples.

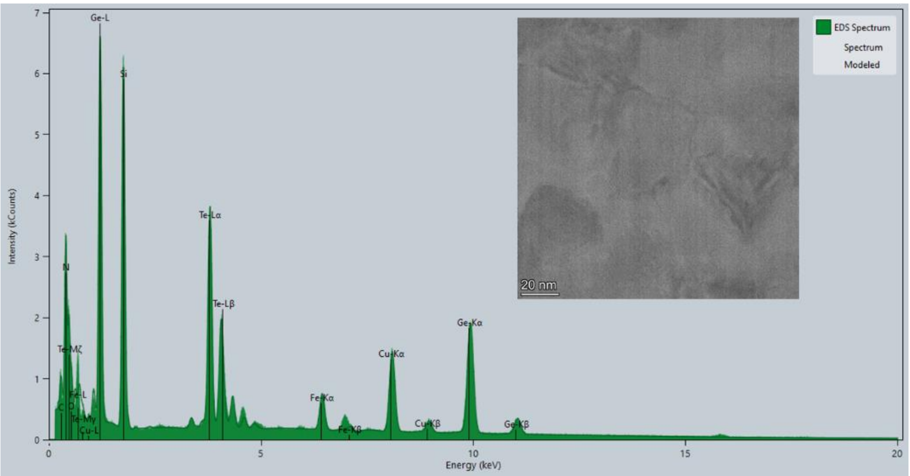

Figure S4: EDX spectrum of the GeTe film, which was deposited on a Si-ntride grid, just after the PLD system had been cleaned. The measured composition of the film is $Ge_{47.2}Te_{52.8}$, which is very close to the composition of the target. The inset shows a corresponding bright field TEM image of the sample.

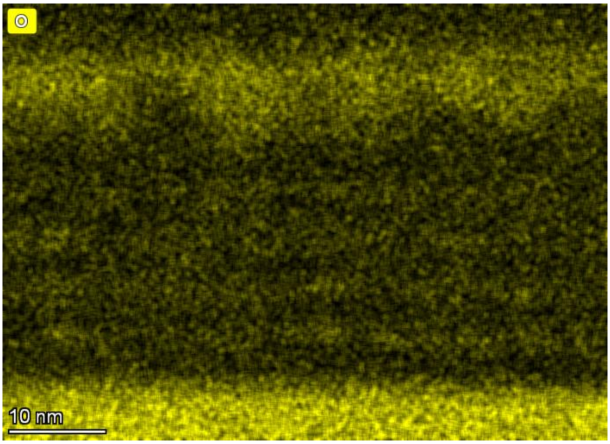

Figure S5: EDX mapping of the element O in the as-deposited thin sample. The layered distribution of O follows the elemental distribution of Ge, as can be observed by comparing this figure with Figure.5e in the main article.

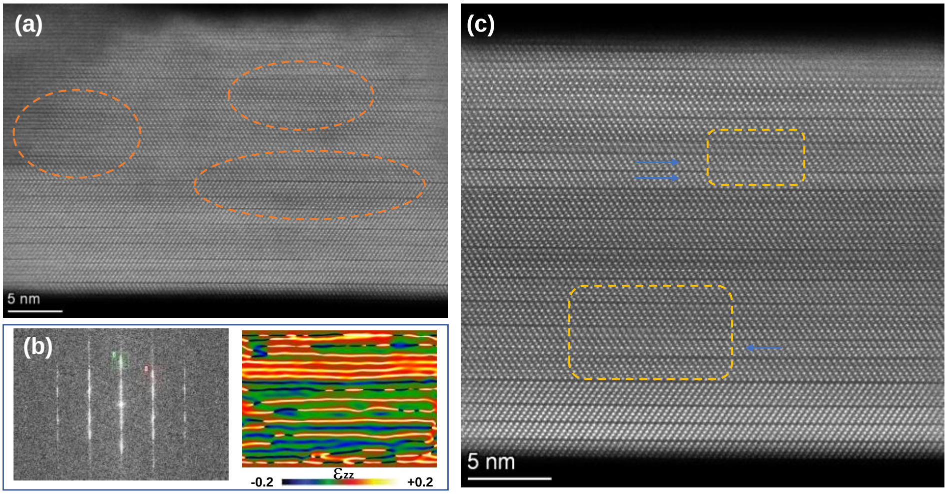

Figure S6: (a) HAADF-STEM image of a fresh 'thin' $Sb_2Te_3/Ge_{1+x}Te$ multilayer after heating once to 210 °C. The orange dotted ellipses show the relative dark areas corresponding to Ge-rich regions. Due to very limited oxidation these areas are not as 'cloudy' as those in the sample stored for one and a half years. Still, the Ge-rich areas can be detected readily. (b) Left: FFT image taken from the HAADF-STEM image (a) of the multilayer, where the squared spots are used for geometric phase analysis (GPA) strain analysis. Right: distribution of the apparent strain in the out-of-plane direction. Obviously, this strain distribution is not homogeneous. The vdW gaps cannot be detected directly through ε_{zz} as the intermixing introduces extra strain in the film. (c) HAADF-STEM image of another region. The blue arrows indicate Sb_2Te_3 quintuple layers, and the neighbouring yellow dotted rectangles show heavily intermixed regions, where quintuple layers dissolve in the $Ge_{1+x}Te$ forming GeSbTe compounds. One should note that this structure is similar to the structure of 'thin' sample after three thermal cycles, demonstrating that the diffusion process is complete and that the $Ge_{1+x}Te$ layers are already saturated after one cycle. This is in good agreement with the thermoelectric property measurements in Figure 4a of the main article.

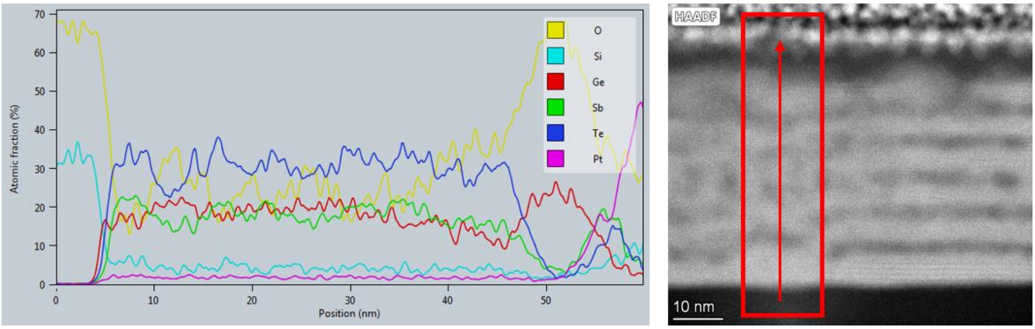

Figure S7: Elemental line profiles (left) of the 'medium sample' after thermal cycling indicating that the oxygen and the Ge concentrations are strongly correlated. The red rectangle and arrow in the HAADF-STEM image (right) show the selected line profile area and direction, respectively.
